# Dynamic Response of CFRP Reinforced Steel Beams Subjected to Impact Action Based on FBG Sensing Technology

**DOI:** 10.3390/s22176377

**Published:** 2022-08-24

**Authors:** Hua-Ping Wang, Yi-Bin Wu, Cong Chen, Hu-Yuan Zhang, Hao Jiang, Xue-Mei Zhang, Xiang-Yang Xu

**Affiliations:** 1 School of Civil Engineering and Mechanics, Lanzhou University, Lanzhou 730000, China; 2 Key Lab of Mechanics on Disaster and Environment in Western China, Lanzhou University, Ministry of Education, Lanzhou 730000, China; 3 Institute of Civil Engineering and Architecture in Gansu Province, Lanzhou 730070, China; 4 School of Rail Transit, Soochow University, Suzhou 215006, China

**Keywords:** CFRP reinforced steel structure, impact action, FBG sensor, dynamic response, time and frequency domain analysis

## Abstract

The in-situ health condition of carbon fiber reinforced polymer (CFRP) reinforced structures has become an important topic, which can reflect the structural performance of the retrofitted structures and judge the design theory. An optical fiber-based structural health monitoring technique is thus suggested. To check the effectiveness of the proposed method, experimental testing on smart CFRP reinforced steel beams under impact action has been performed, and the dynamic response of the structure has been measured by the packaged FBG sensors attached to the surface of the beam and the FBG sensors inserted in the CFRP plates. Time and frequency domain analysis has been conducted to check the structural feature of the structures and the performance of the installed sensors. Results indicate that the packaged Fiber Bragg Grating (FBG) sensors show better sensing performance than the bare FBG sensors in perceiving the impact response of the beam. The sensors embedded in the CFRP plate show good measurement accuracy in sensing the external excitation and can replace the surface-attached FBG sensors. The dynamic performance of the reinforced structures subjected to the impact action can be straightforwardly read from the signals of FBG sensors. The larger impact energies bring about stronger impact signals.

## 1. Introduction

Carbon fiber reinforced polymer (CFRP) composites have been extensively used in strengthening projects due to their high strength, lightweight, corrosion resistance, and design flexibility. CFRP-reinforced structures have become the commonly used structural type in engineering [1,2,3]. Impact action is a common type of loading on steel structures (i.e., bridges, off-shore platforms, railway tracks, and buildings). For example, CFRP reinforced steel or concrete beam structures can be the major components of existing bridges due to the rehabilitation design. The moving vehicles may act impact action on this kind of structure. Therefore, it is important to understand the structural response of CFRP reinforced structures under impact action, which can be used to assess the effectiveness of the retrofit strategies, instruct the impact-resistant design, and improve the performance of reinforced structures.

Considerable research has contributed to exploring the dynamic performance of CFRP reinforced structures [4]. Zanardo et al. [5] studied the dynamic assessment method based on vibration tests and modal analysis to determine the elastic flexural stiffness of bridge structures retrofitted with CFRP. Capozucca [6,7,8] identified the damage degree of reinforced concrete (RC) structures with CFRP plates by the variations of natural frequency and vibration mode shapes recorded during the tests. Radnić et al. [9] explored the dynamic response of reinforced concrete (RC) slabs with CFRP strips under impact load through experiments and simulation. Prado et al. [10] conducted the modal analysis to check the shear force-induced damage in CFRP reinforced beams. Liu and Xiao [11] performed impact testing and found that the reaction force rather than the impact force should be used to reflect the impact resistance. Alam et al. [12] studied the effect of impact energy on the lateral displacement control ability of FRP-strengthened concrete-filled steel tubular (CFST) members. Salvetti et al. [13] proposed an improved model based on Olsson’s work to predict the dynamic behavior of CFRP plates under low-velocity impact. Li et al. [14,15] used modal analysis and regression data to formulate constitutive equations to describe the dynamic bond-slip behavior of the CFRP–concrete interface. Liu et al. [16] studied the dynamic impact-resistant properties of wood beams reinforced with CFRP. Generally, experimental testing and finite element analysis have been used to explore the response of CFRP-reinforced structures under impact action, which aims to check the differences between the actual performance and the design value. It can be noted that limitations exist in special case studies due to being conducted in laboratory conditions. For this reason, structural health monitoring is suggested to check the dynamic response of CFRP reinforced structures [17,18], which can provide real-time and long-term data for scientific analysis.

Due to the superior advantages of absolute measurement, anti-electromagnetic interference, good geometrical shape-versatility, high precision, compact size, lightweight, convenient multiplexing, and integration of sensing network over other sensors [19,20,21,22,23,24], optical fiber sensing technology has thus been suggested to perform the health monitoring of CFRP assembled structures under static and dynamic loads. The assembly of CFRP composites with optical fiber sensing elements to configure the smart CFRP components has also been performed by some scholars [18]. However, the dynamic performance of the CFRP reinforced structures installed with optical fiber-based sensors has seldom been explored. Further study is still required to explore the effectiveness and feasibility of the proposed monitoring technique to assess the dynamic response.

Given the analysis above, the dynamic response of steel beam retrofitted by smart CFRP plates under impact action has been explored by the surface-attached packaged Fiber Bragg Grating (FBG) sensors and the FBG sensors inserted in the CFRP plates. Impact testing with different impact energies has been performed to check the performance of the reinforced structures. Time and frequency domain analysis has been conducted to assess the performance of the structures and the sensors. Based on the data analysis, a few suggestions on the sensor design and the reinforced structures have been provided.

## 2. Experimental Model

**Figure 1:**
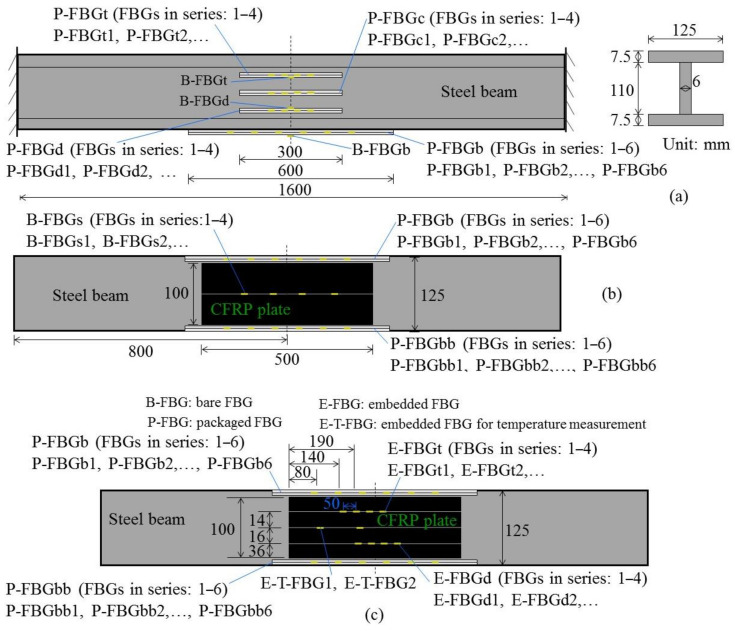
Layout of FBG sensors on the testing sample: bare and packaged FBG sensors (**a**) on the web and (**b**) beam bottom of the I-steel beam; (**c**) FBG sensors embedded in the CFRP plate.

To explore the dynamic response of CFRP reinforced steel beams under impact action, a testing sample installed with various kinds of FBG sensors has been fabricated. The testing sample consists of one I-steel beam reinforced by a smart CFRP plate. The cross section of the I-steel beam is 125 × 125 mm^2^, and the length of the beam is 1600 mm. The material properties of the steel beam obey the standard GB/T 11263-2017. Packaged FBG sensors and bare FBG sensors have been attached to the surface of the smart CFRP reinforced steel beam, and the layout is shown in Figure 1a,b. The layout of the FBG sensors embedded in the smart CFRP plate follows Figure 1c. FBG sensors have been adhered to the surface of the steel beam by the mixture composed of Dow Corning SYLGARD™ 184 Silicone Elastomer and the correlated curing agent. The CFRP plate have been adhered to the beam bottom of the steel beam by an epoxy resin mixture [25]. There are 14 FBG sensors arranged on the web of the beam, numbered P-FBGt (FBGs in series: 1–4), P-FBGc (FBGs in series: 1–4), P-FBGd (FBGs in series: 1–4), B-FBGt and B-FBGd. In total, 17 FBG sensors are attached on the beam bottom, numbered P-FBGb (FBGs in series: 1–6), P-FBGbb (FBGs in series: 1–6), B-FBGs (FBGs in series: 1–4), and B-FBGb. There are 10 FBG sensors embedded in the CFRP plate, numbered E-FBGt (FBGs in series: 1–4), E-FBGd (FBGs in series: 1–4), E-T-FBG1 and E-T-FBG2. P-FBG means the flexible silicone rubber packaged FBG. B-FBG means bare FBG. E-FBG means the embedded FBG. E-T-FBG means the embedded FBG without constraint just used for temperature measurement [26,27].

**Figure 2:**
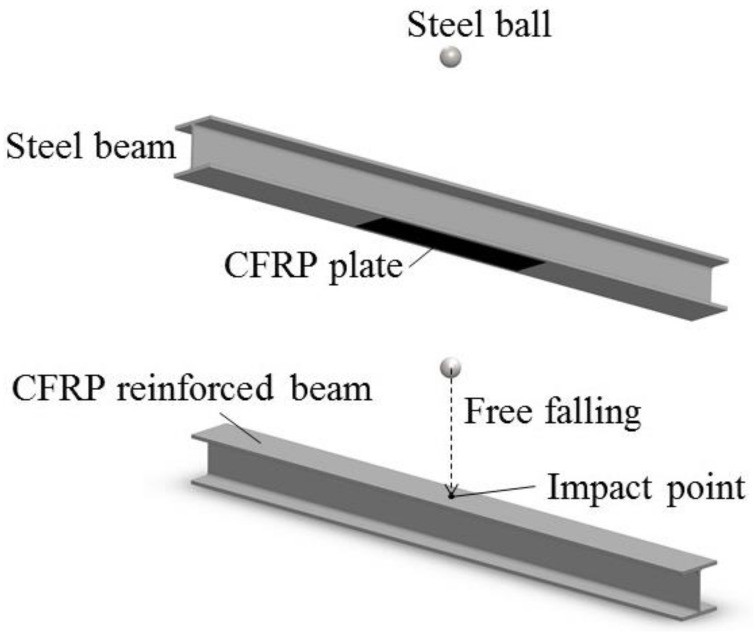
Implementation of the impact action by steel ball.

**Figure 3:**
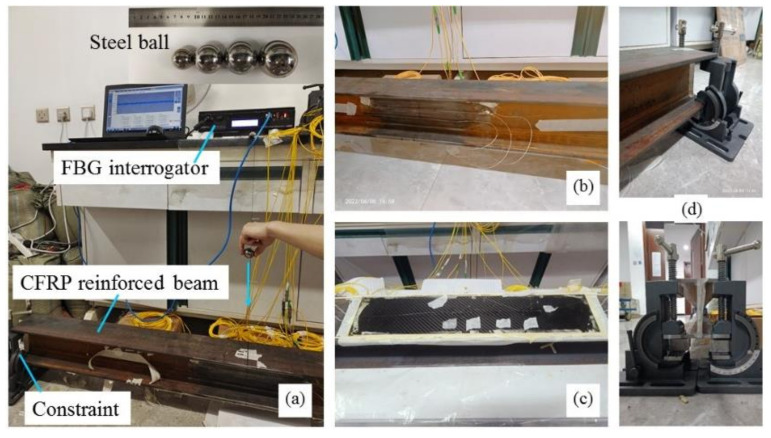
(**a**) Experimental device of the CFRP reinforced steel beam subjected to impact load; physical photo of FBG sensors attached on (**b**) the web and (**c**) beam bottom of the beam; (**d**) clamped boundary condition.

All the FBG sensors have been connected to FBG interrogator si255 produced by Micron Optics (MOI), which has a sampling frequency of 5 kHz and a wavelength resolution of 1 pm. The impact action is provided by the free falling of steel balls with different diameters at different heights. Fixed constraints have been applied to the two ends of the beam by means of clamps. Four heights (20 cm, 30 cm, 40 cm, 50 cm) and four weights of steel balls (110.12 g, 261.02 g, 376.55 g, 508.50 g) have been tried in the testing, and the cases for the impact action are shown in Table 1. The impact point locates in the midspan of the upper wing panel of the CFRP reinforced beam, and the implementation of the impact action by the steel ball is illustrated in Figure 2. The experimental device designed for studying the dynamic response of the CFRP reinforced beam under impact loading is shown in Figure 3. During each impact testing, the FBG interrogator and the connected computer automatically record the wavelength changes of each FBG sensor. The dynamic characteristics of the structure can be obtained by analyzing the impact response of the CFRP reinforced steel beam under various impact conditions. In reference to the impact energy, when the impact height and the ball weight are given, the impact energy can be calculated by Equation (1).

(1) $$E_{I}=mgh$$

where m is the weight of the steel ball, g is the acceleration of gravity, h is the impact height, $E_{I}$ is the impact energy.

## 3. Time Domain Analysis

### 3.1. FBG Sensors Attached on the Web of the Beam

**Figure 4:**
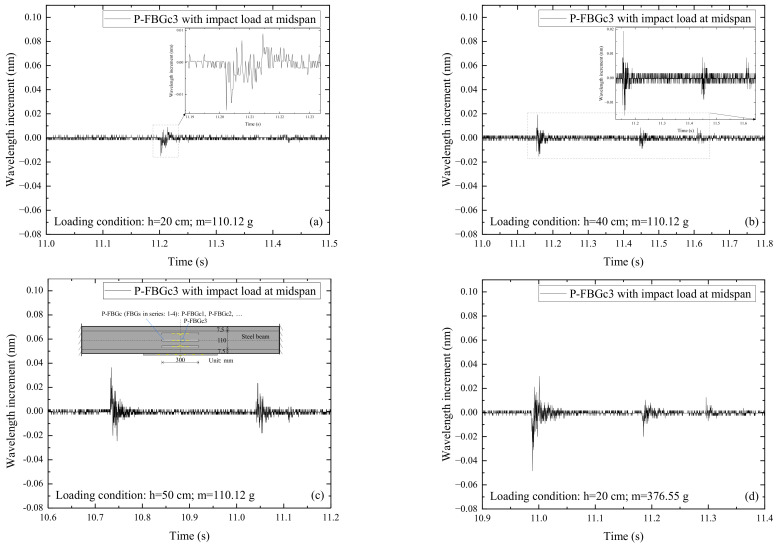

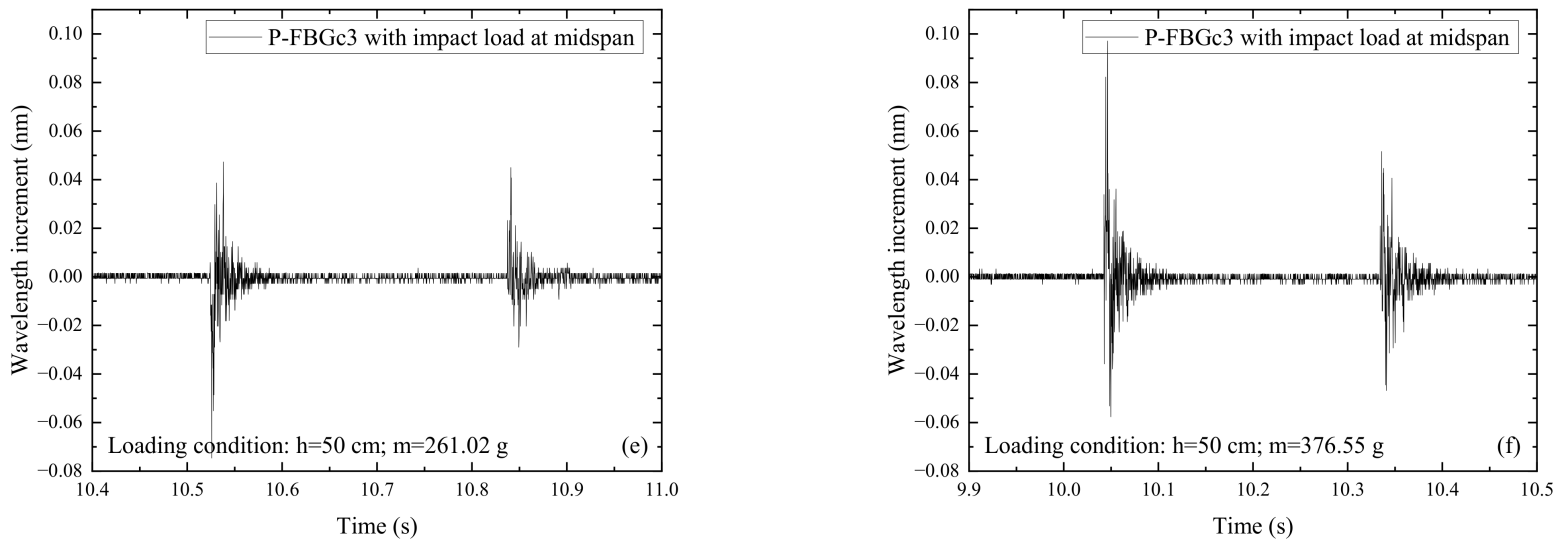
Signals of P-FBGc3 when the CFRP reinforced steel beam is subjected to the impact action induced by the steel ball with different weights (m) and falling heights (h): (**a**) *h* = 20 cm, *m* = 110.12 g; (**b**) *h* = 40 cm, *m* = 110.12 g; (**c**) *h* = 50 cm, *m* = 110.12 g; (**d**) *h* = 20 cm, *m* = 376.55 g; (**e**) *h* = 50 cm, *m* = 261.02 g; (**f**) *h* = 50 cm, *m* = 376.55 g.

It has been found from the wavelength incremental diagrams that the FBG signals under various impact conditions have similar waveforms. Figure 4 shows the signals of P-FBGc3 when the CFRP reinforced steel beam is subjected to the impact action induced by the steel ball with different weights (m) and falling heights (h). It can be seen from Figure 4a that when the impact height and ball weight are small, the wavelength increment of the FBG reaches a maximum at the moment of impact, then rapidly decays and oscillates at a stable value. The FBG signals in this condition show multiple peaks, but only a single peak is obtained due to the signal noise. From Figure 4b–f, it can be seen that when the impact height and ball weight are large, multiple peaks appear in the FBG signals. The wavelength increments reach a maximum at the moment of impact, then rapidly decay and oscillate smoothly. Because the free-falling steel ball impacts the CFRP reinforced steel beam several times, the smooth wavelength increments show multiple peaks. However, due to the energy loss of the steel ball, the following peaks are smaller than the peak at the first impact. In general, the amplitude of the wavelength increments of the FBG on the web increases with the growth of the impact energy.

**Figure 5:**
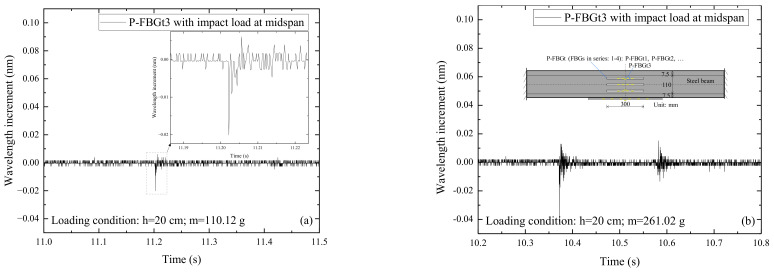

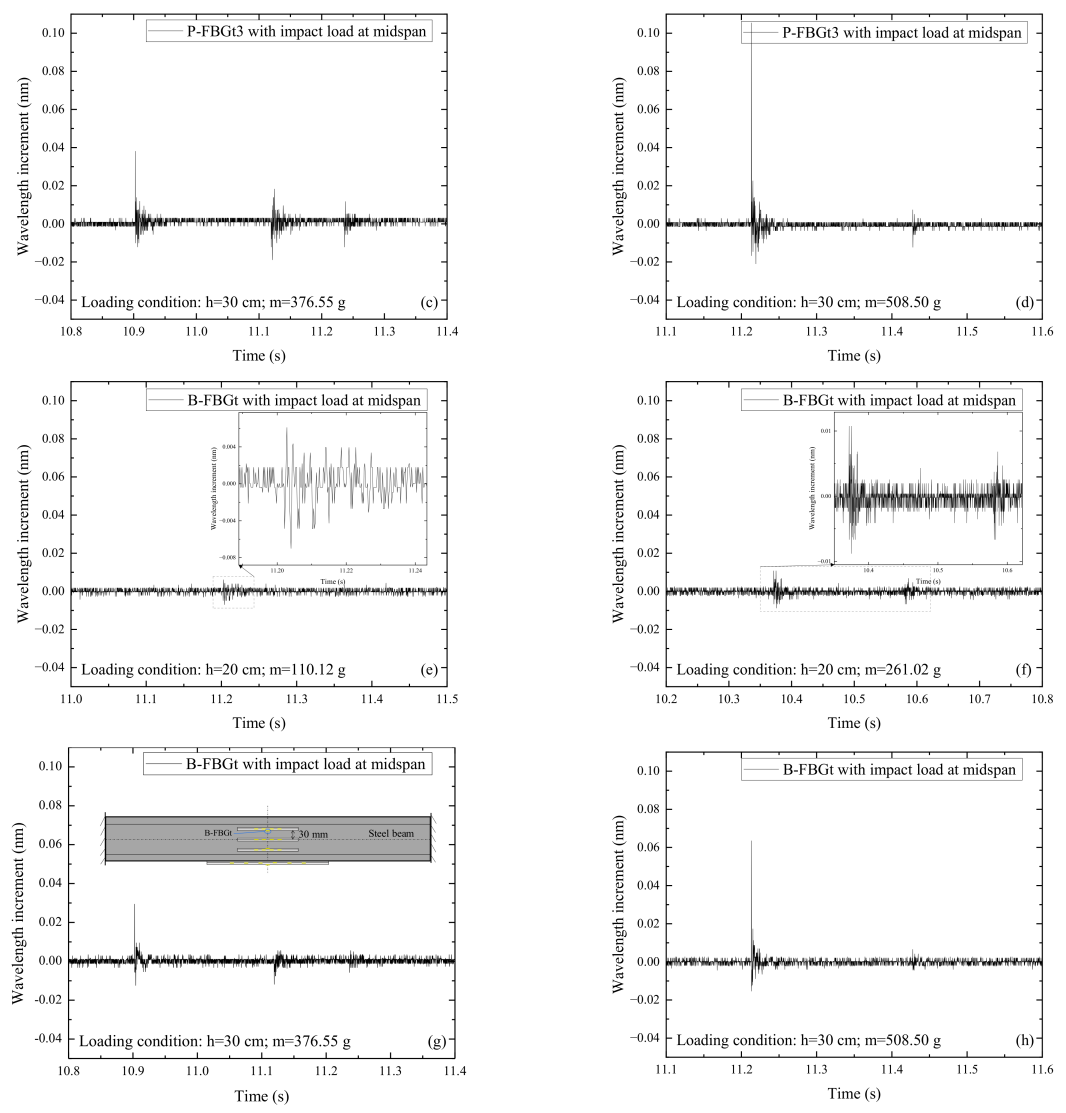
Signals of P-FBGt3 and B-FBGt when the CFRP reinforced steel beam is subjected to the impact action induced by the steel ball with different weights (m) and falling heights (h): (**a**) P-FBGt3, *h* = 20 cm, *m* = 110.12 g; (**b**) P-FBGt3, *h* = 20 cm, *m* = 261.02 g; (**c**) P-FBGt3, *h* = 30 cm, *m* = 376.55 g; (**d**) P-FBGt3, *h* = 30 cm, *m* = 508.50 g; (**e**) B-FBGt, *h* = 20 cm, *m* = 110.12 g; (**f**) B-FBGt, *h* = 20 cm, *m* = 261. 02 g; (**g**) B-FBGt, *h* = 30 cm, *m* = 376.55 g; (**h**) B-FBGt, *h* = 30 cm, *m* = 508.50 g.

Figure 5 shows the signals of P-FBGt3 and B-FBGt when the CFRP reinforced steel beam is subjected to the impact action induced by the steel ball with different weights (m) and falling heights (h). Among them, P-FBGt3 is the packaged FBG attached to the web, and B-FBGt is the bare FBG attached to the web. Comparing Figure 5a,b with Figure 5e,f, it can be seen that the impact signal of P-FBGt3 is more pronounced than that of B-FBGt when the impact height and steel ball weight are small. It indicates that the packaged FBG attached on the web is more effective in monitoring the external excitation on the CFRP reinforced steel beam. The reason may be that the overall deformation coordination of the packaging material is better, while the bare FBG with a small size is limited to perceive the external excitation sensitively. Comparing Figure 5c,d with Figure 5g,h, it can be seen that the impact signal of P-FBGt3 is still more obvious than that of B-FBGt when the impact height and ball weight are larger. Multiple peaks are obvious in the P-FBGt3 signal, and multiple peaks in the B-FBGt signal are less obvious due to the influence of signal noise. Overall, the packaged FBG sensors attached on the web are much more sensitive to the dynamic response than that of the bare FBG sensors, which can play a better role in monitoring dynamic structural features.

### 3.2. FBG Sensors Attached on the Beam Bottom of the Beam

**Figure 6:**
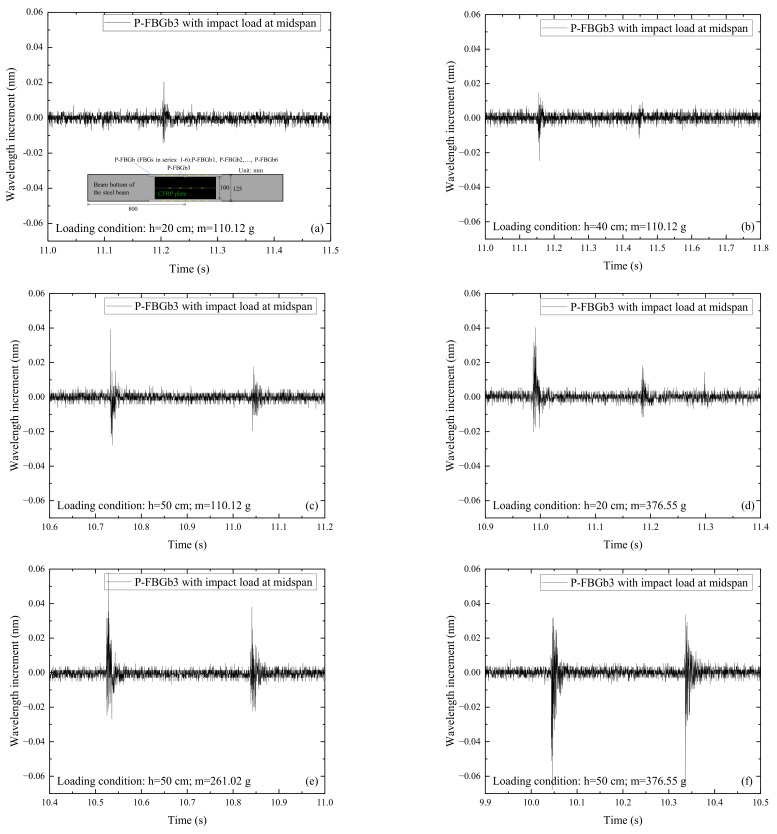
Signals of P-FBGb3 when the CFRP reinforced steel beam is subjected to the impact action induced by the steel ball with different weights (m) and falling heights (h): (**a**) *h* = 20 cm, *m* = 110.12 g; (**b**) *h* = 40 cm, *m* = 110.12 g; (**c**) *h* = 50 cm, *m* = 110.12 g; (**d**) *h* = 20 cm, *m* = 376.55 g; (**e**) *h* = 50 cm, *m* = 261.02 g; (**f**) *h* = 50 cm, *m* = 376.55 g.

Similar to the time domain analysis of the measured signals on the web, it has been found from the wavelength incremental diagrams that the FBG signals under various impact conditions have similar waveforms. Figure 6 shows the signals of P-FBGb3 when the CFRP reinforced steel beam is subjected to the impact action induced by the steel ball with different weights (m) and falling heights (h). It can be seen from Figure 6a that when the impact height and ball weight are small, the wavelength increment of the FBG reaches a maximum at the moment of impact, then rapidly decays and oscillates at a stable value. The FBG signals in this condition show multiple peaks, but only a single peak is obtained due to the signal noise. It can be seen from Figure 6b–f that when the impact height and ball weight are large, multiple peaks appear in the FBG signals, and the wavelength increments reach a maximum at the moment of impact, and then rapidly decay. Because the free-falling steel ball impacts the CFRP reinforced steel beam several times, the wavelength increments have multiple peaks. However, due to the energy loss of steel ball, the peak value is smaller than that at the previous impact. In addition, the peaks show that the number of impact actions of the free-falling steel ball on the CFRP reinforced steel beam is two to three. Overall, the amplitude of the wavelength increment of the FBG on the bottom plate increases with the growth of the impact energy.

**Figure 7:**
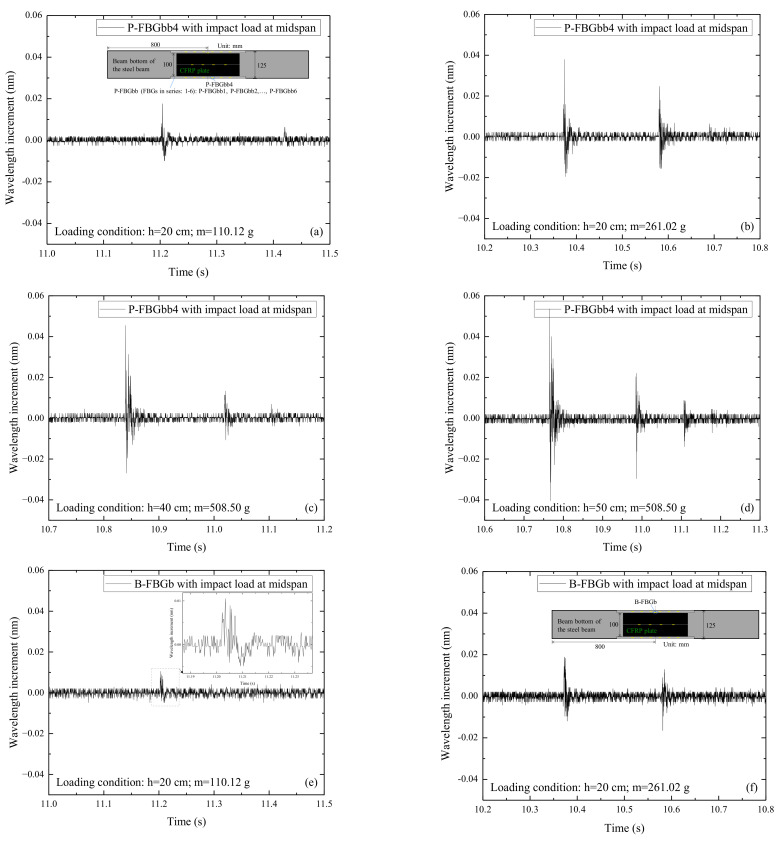

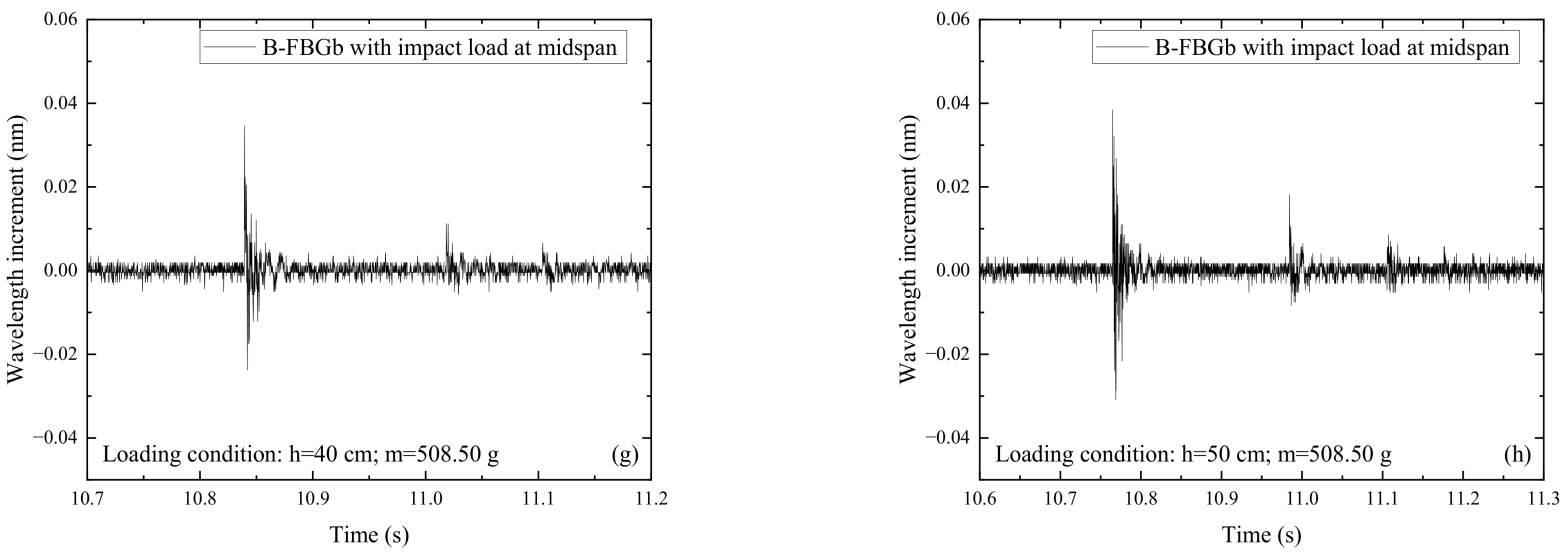
Signals of P-FBGbb4 and B-FBGb when the CFRP reinforced steel beam is subjected to the impact action induced by the steel ball with different weights (m) and falling heights (h): (**a**) P-FBGbb4, *h* = 20 cm, *m* = 110.12 g; (**b**) P-FBGbb4, *h* = 20 cm, *m* = 261.02 g; (**c**) P-FBGbb4, *h* = 40 cm, *m* = 508.50 g; (**d**) P-FBGbb4, *h* = 50 cm, *m* = 508.50 g; (**e**) B-FBGb, *h* = 20 cm, *m* = 110.12 g; (**f**) B-FBGb, *h* = 20 cm, *m* = 261. 02 g; (**g**) B-FBGb, *h* = 40 cm, *m* = 508.50 g; (**h**) B-FBGb, *h* = 50 cm, *m* = 508.50 g.

Figure 7 shows the signals of P-FBGbb4 and B-FBGb when the CFRP reinforced steel beam is subjected to the impact action induced by the steel ball with different weights (m) and falling heights (h). Among them, P-FBGbb4 is the packaged FBG, and B-FBGb is the bare FBG attached to the beam bottom. Comparing Figure 7a,b with Figure 7e,f, it can be seen that the impact signal of P-FBGbb4 is more obvious than B-FBGb when the impact height and steel ball weight are small. It indicates that the packaged FBG is more effective in monitoring the external excitation-induced dynamic response of the CFRP reinforced steel beam. The reason may be that the overall deformation coordination of the packaging material is better, while the bare FBG with a small size is limited to sensitively perceive the external excitation. Comparing Figure 7c,d with Figure 7g,h, it can be seen that the impact signal of P-FBGbb4 is still more obvious than that of B-FBGb when the impact height and steel ball weight are larger. Overall, the monitoring effect of the packaged FBG is better than the bare FBG attached to the beam bottom.

### 3.3. FBG Sensors Embedded in the CFRP Plate

**Figure 8:**
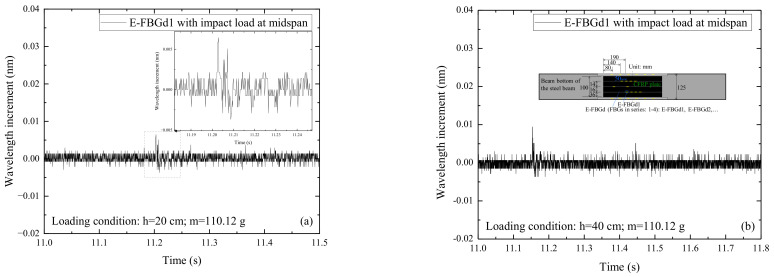

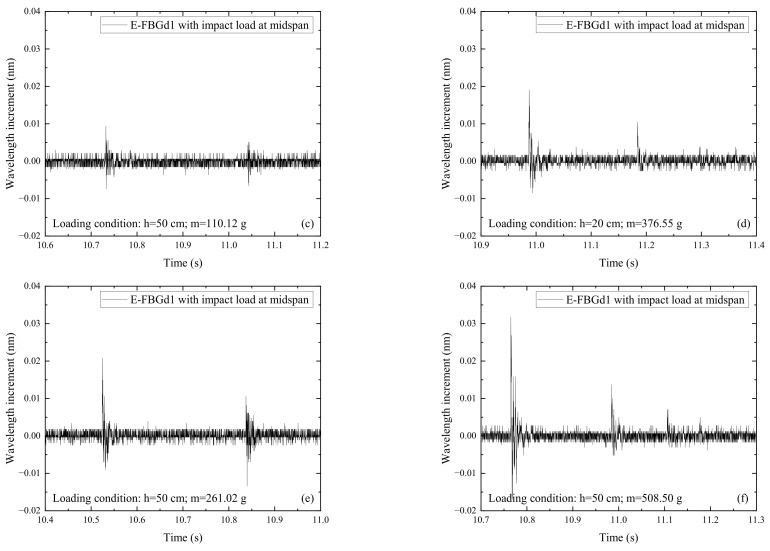
Signals of E-FBGd1 when the CFRP reinforced steel beam is subjected to the impact action induced by the steel ball with different weights (m) and falling heights (h): (**a**) *h* = 20 cm, *m* = 110.12 g; (**b**) *h* = 40 cm, *m* = 110.12 g; (**c**) *h* = 50 cm, *m* = 110.12 g; (**d**) *h* = 20 cm, *m* = 376.55 g; (**e**) *h* = 50 cm, *m* = 261.02 g; (**f**) *h* = 50 cm, *m* = 508.50 g.

Wavelength increment diagrams of the embedded FBG of the CFRP plates under various impact conditions have similar waveforms. Figure 8 shows the signals of E-FBGd1 when the CFRP reinforced steel beam is subjected to the impact action induced by the steel ball with different weights (m) and falling heights (h). The wavelength increment amplitude of the FBG sensors embedded in the CFRP plate increases with the growth of the impact energy. Comparing Figure 4 and Figure 6 with Figure 8, it can be seen that the signals of FBG sensors embedded in the CFRP plate are generally weaker than that of the FBG sensors attached to the web or beam bottom but still well senses the external excitation.

**Figure 9:**
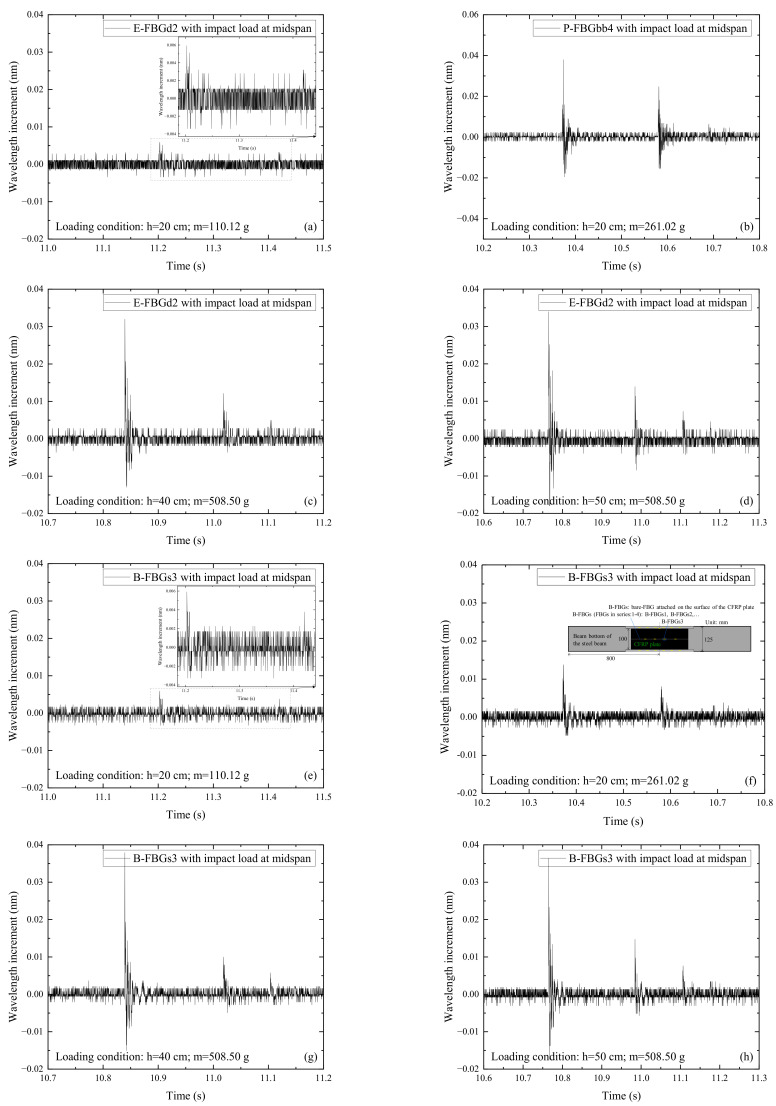
Signals of E-FBGd2 and B-FBGs3 when the CFRP reinforced steel beam is subjected to the impact action induced by the steel ball with different weights (m) and falling heights (h): (**a**) E-FBGd2, *h* = 20 cm, *m* = 110.12 g; (**b**) P-FBGbb4, *h* = 20 cm, *m* = 261.02 g; (**c**) E-FBGd2, *h* = 40 cm, *m* = 508.50 g; (**d**) E-FBGd2, *h* = 50 cm, *m* = 508.50 g; (**e**) B-FBGs3, *h* = 20 cm, *m* = 110.12 g; (**f**) B-FBGs3, *h* = 20 cm, *m* = 261.02 g; (**g**) B-FBGs3, *h* = 40 cm, *m* = 508.50 g; (**h**) B-FBGs3, *h* = 50 cm, *m* = 508.50 g.

Figure 9 shows the signals of E-FBGd2 and B-FBGs3 when the CFRP reinforced steel beam is subjected to the impact action induced by the steel ball with different weights (m) and falling heights (h). E-FBGd2 is the FBG embedded in the CFRP plate, and B-FBGs3 is the bare FBG attached to the surface of the CFRP plate. Comparing Figure 9a,b with Figure 9e,f, it can be seen that the impact signal of E-FBGd2 is similar to that of B-FBGs3 when the impact height and ball weight are small. Comparing Figure 9c,d with Figure 9g,h, it can be seen that the impact signal of E-FBGd2 is still similar to that of B-FBGs3 when the impact height and ball weight are larger. It indicates that the embedded FBG sensors can replace the surface attached sensors to perceive the impact response of the CFRP plate. Overall, the monitoring effect of FBG sensors embedded in the CFRP plate is equivalent to that of the bare FBG attached to the surface of the CFRP plate. However, the embedded FBG has the advantage of being able to survive in a harsh working environment due to the protection of the CFRP composites.

**Figure 10:**
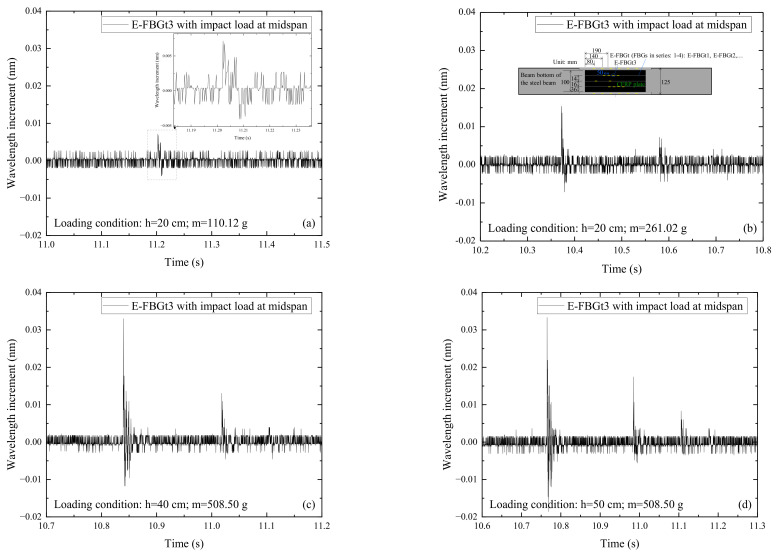

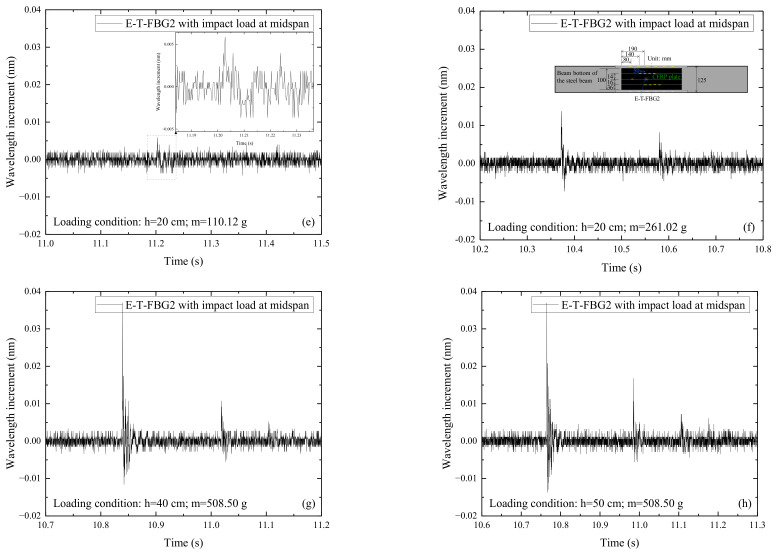
Signals of E-FBGt3 and E-T-FBG2 when the CFRP reinforced steel beam is subjected to the impact action induced by the steel ball with different weights (m) and falling heights (h): (**a**) E-FBGt3, *h* = 20 cm, *m* = 110.12 g; (**b**) E-FBGt3, *h* = 20 cm, *m* = 261.02 g; (**c**) E-FBGt3, *h* = 40 cm, *m* = 508.50 g; (**d**) E-FBGt3, *h* = 50 cm, *m* = 508.50 g; (**e**) E-T-FBG2, *h* = 20 cm, *m* = 110.12 g; (**f**) E-T-FBG2, *h* = 20 cm, *m* = 261.02 g; (**g**) E-T-FBG2, *h* = 40 cm, *m* = 508.50 g; (**h**) E-T-FBG2, *h* = 50 cm, *m* = 508.50 g.

Figure 10 shows the signals of E-FBGt3 and E-T-FBG2 when the CFRP reinforced steel beam is subjected to the impact action induced by the steel ball with different weights (m) and falling heights (h). E-FBGt3 is the FBG embedded in the CFRP plate, and E-T-FBG2 is the free FBG inside the CFRP plate, which is just used for temperature measurement. Comparing Figure 10a,b with Figure 10e,f, it can be seen that when the impact height and ball weight are small, E-T-FBG2 produces impact signals, and the peak value is similar to that of E-FBGt3. Comparing Figure 10c,d with Figure 10g,h, it can be seen that E-T-FBG2 still produces impact signals, and the peak value is similar to that of E-FBGt3 when the impact height and ball weight are larger. The reason may be that the unconstrained E-T-FBG2 under the impact load is subjected to a small dynamic response induced by the frictional effect of the deformed casing pipe wall. It indicates that the sensor just used for temperature measurement cannot be embedded in the structure with a hollow pipe, which is different from the static case. For the temperature compensation of structures under dynamic load, it is suggested that the sensor for temperature measurement should be around the structure.

## 4. Frequency Domain Analysis

Time domain analysis shows that the FBG sensor can accurately monitor the dynamic response signal. To identify the dynamic characteristics of the CFRP reinforced steel beam under the impact action, frequency domain analysis is further required [28,29]. Because the power spectrum is commonly used for frequency domain analysis of the nonstationary signal, the power spectrum is adopted for frequency domain analysis of the measured signals [30,31]. The equation of the discrete Fourier transform is shown in Equation (2):

(2) $$X\left[k\right]=\sum_{n=0}^{N-1}x\left[n\right]e^{-k\frac{2\pi ni}{N}},k=0,1,\ldots ,N-1$$

where $X\left[k\right]$ is the frequency domain distribution of the signal, and $x\left[n\right]$ is the time domain distribution of the signal.

The equation for power spectrum estimation by the periodogram method is shown in Equation (3):

(3) $$I_{N}(k)={\left.I_{N}(\omega )\right|}_{\omega =\frac{2\pi }{N}k}=\frac{1}{N}|X(k){|}^{2},\;k=0,1,\ldots ,N-1$$

where X(k) is the Fourier variation of x(k), k=0,1,…,N−1 of the time domain signal, and $I_{N}(k)$ is the power spectrum estimate of the signal. In this paper, the modified periodogram method with Hamming window is used to find the power spectrum of the FBG signal under each impact condition. When the impact energy is relatively small, the direct current (DC) component of the FBG signal in the frequency domain is much more obvious, resulting in the peak of the high-frequency component being extremely insignificant and challenging to be identified. Therefore, during the frequency domain analysis, some cases with minimal impact energy will be ignored.

### 4.1. FBG Sensors Attached on the Web of the Beam

**Figure 11:**
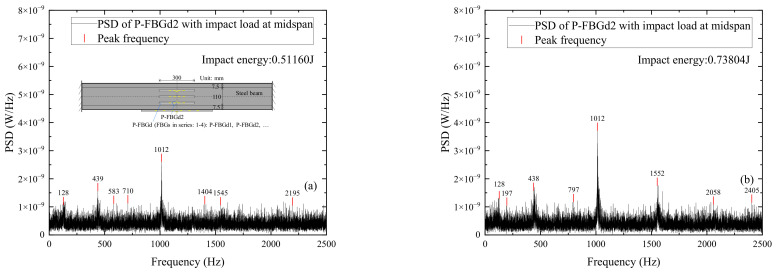

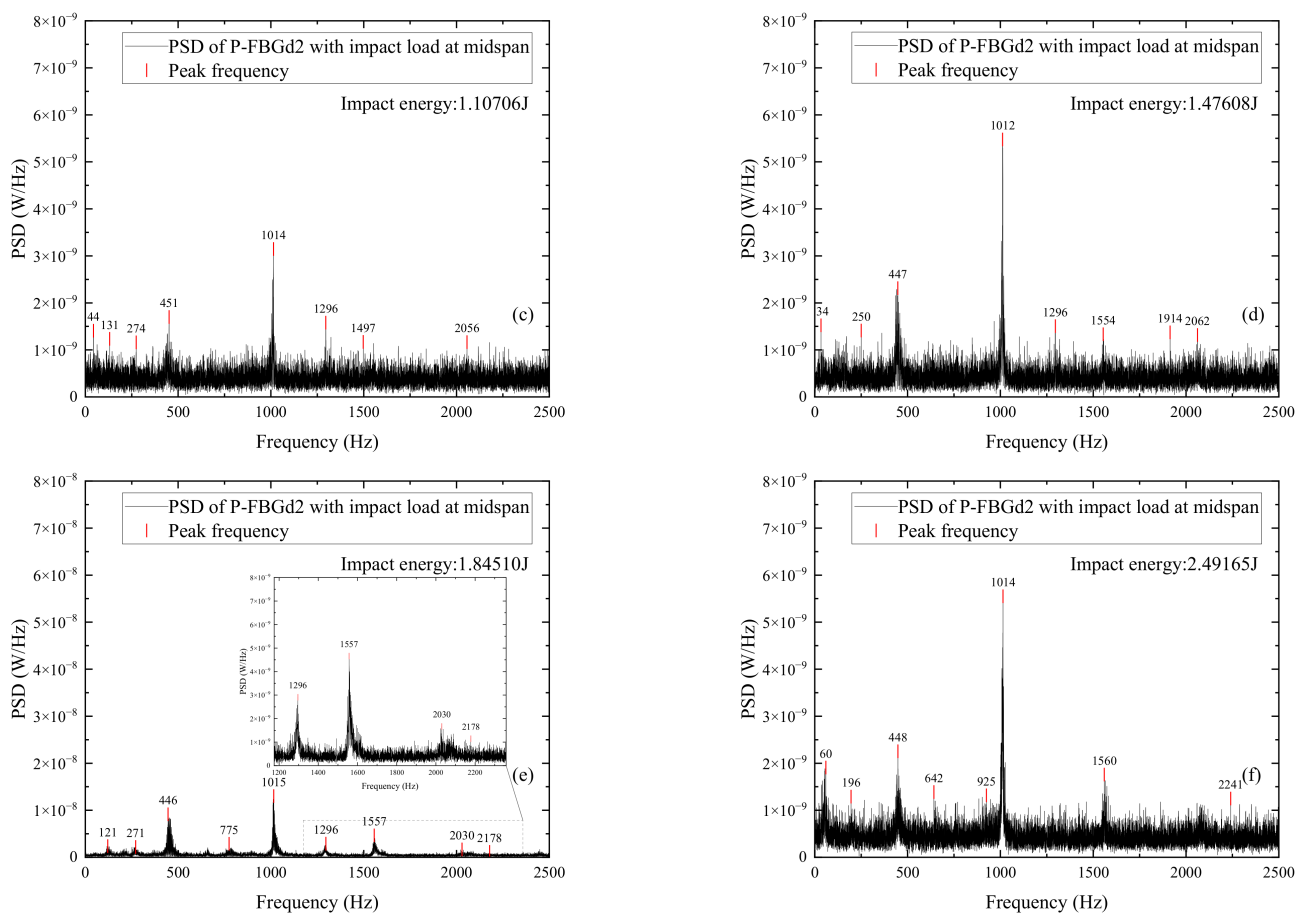
Power spectral density (PSD) distributions of signals measured by P-FBGd2 when the CFRP reinforced beam is subjected to different impact energies: (**a**) 0.51160 J; (**b**) 0.73804 J; (**c**) 1.10706 J; (**d**) 1.47608 J; (**e**) 1.84510 J; (**f**) 2.49165 J.

Figure 11 shows the power spectrum distributions of signals measured by P-FBGd2 when the CFRP reinforced beam is subjected to different impact energies. It can be seen From Figure 11a–c that the peak value of FBG signals is less pronounced when the impact energy is small [32,33]. However, it can be seen from Figure 11d–f that when the impact energy is large, the peak value of FBG signals is prominent and can be clearly identified. In addition, a comparison of the power spectrum at various impact energies in Figure 11 also shows that although the peak values of the power spectrum at different impact energies vary, the peak frequencies at each impact condition basically match (i.e., peak frequencies around 440 Hz, 1000 Hz, 1550 Hz, and 2200 Hz), indicating that no damage exists in the CFRP reinforced beam at each impact condition. Some extra peak frequencies in individual diagrams may be due to the differences in the impact action of the free-falling steel ball.

**Figure 12:**
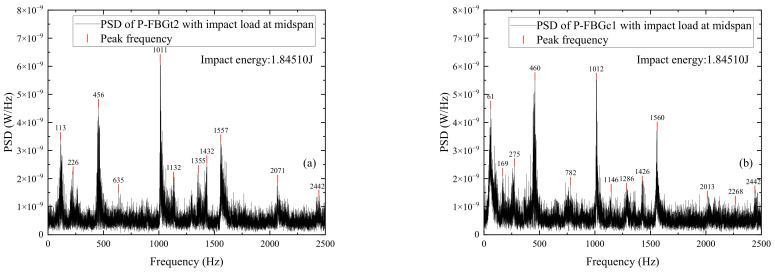

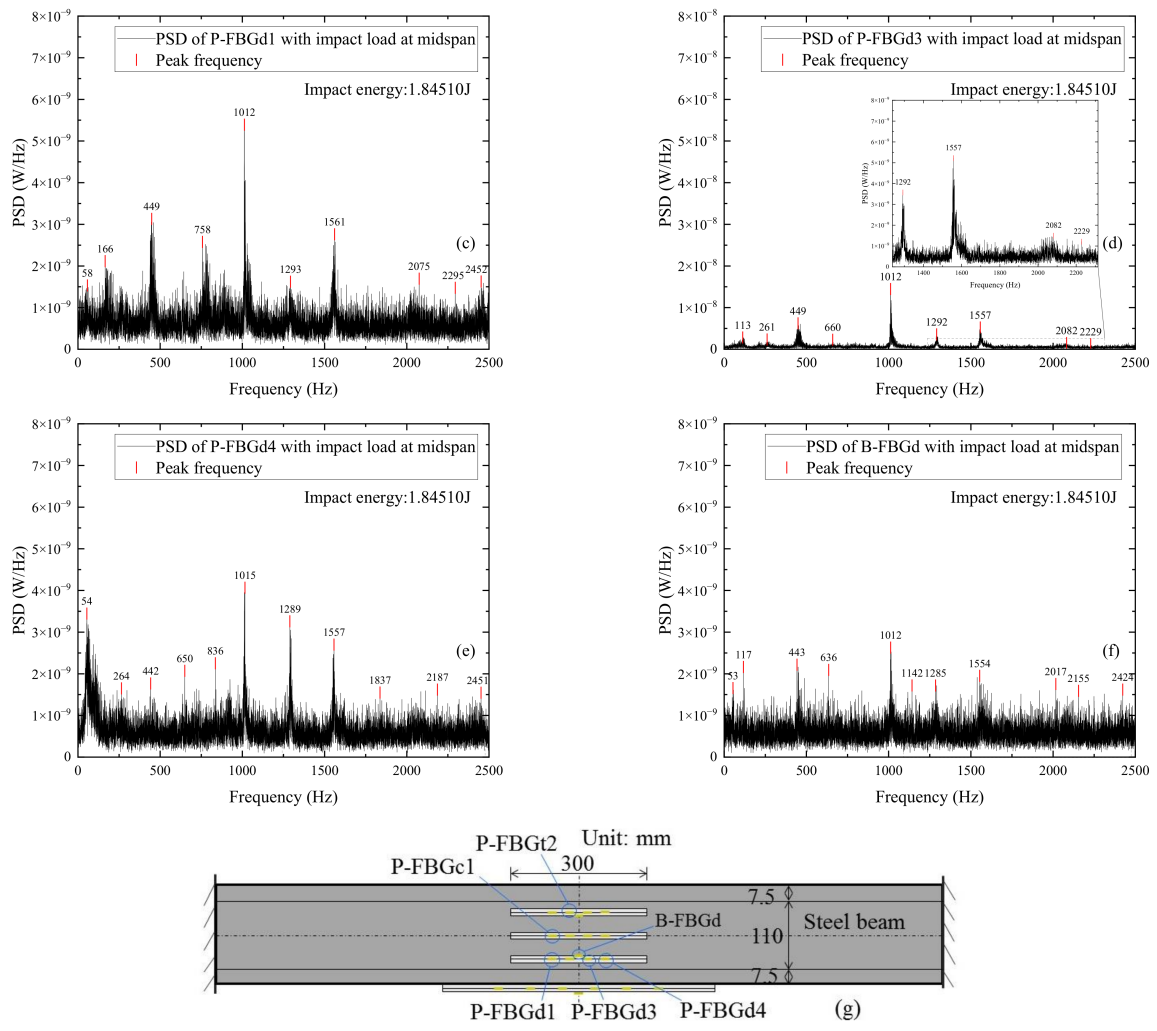
PSD distributions of signals measured by different FBG sensors when the CFRP reinforced beam is subjected to impact energy 1.8451 J: (**a**) P-FBGt2; (**b**) P-FBGc1; (**c**) P-FBGd1; (**d**) P-FBGd3; (**e**) P-FBGd4; (**f**) B-FBGd; (**g**) Locations of the 6 FBGs.

Figure 12 shows the power spectrum distributions of signals measured by different FBG sensors when the CFRP reinforced beam is subjected to impact energy 1.8451 J. The sensors include P-FBGt2, P-FBGc1, P-FBGd1, P-FBGd3, P-FBGd4, and B-FBGd attached on the web. Comparing Figure 12a,b with Figure 12c–e, it can be seen that the peak signal of the FBG sensor closer to the impact point is more pronounced when the impact energy is the same. Comparing Figure 12a–e with Figure 12f, it can be seen that the peak frequencies of the packaged FBG are basically the same as that of the bare FBG when the impact energy is the same. Since the position of P-FBGd3 is closer to that of B-FBGd, a comparison in Figure 12d,f is conducted. It shows that when the impact energy is the same, the peak signal of P-FBGd3 is significantly higher than that of B-FBGd, indicating that the packaged FBG identifies the impact signal better than the bare FBG.

### 4.2. FBG Sensors Attached on the Beam Bottom of the Beam

It is found from the power spectrum that the impact signals of P-FBGb (FBGs in series: 1–6) on the beam bottom are less pronounced in either impact condition. The reason may be that the packaged sensor has not been well attached to the CFRP reinforced beam due to the poor installation quality. Another sensor P-FBGbb (FBGs in series: 1–6) also located at the beam bottom shows good sensitivity to the impact signal. Therefore, the frequency domain analysis of the measured signal on the beam bottom will focus on P-FBGb2, P-FBGbb (FBGs in series: 1–6) and B-FBGb.

**Figure 13:**
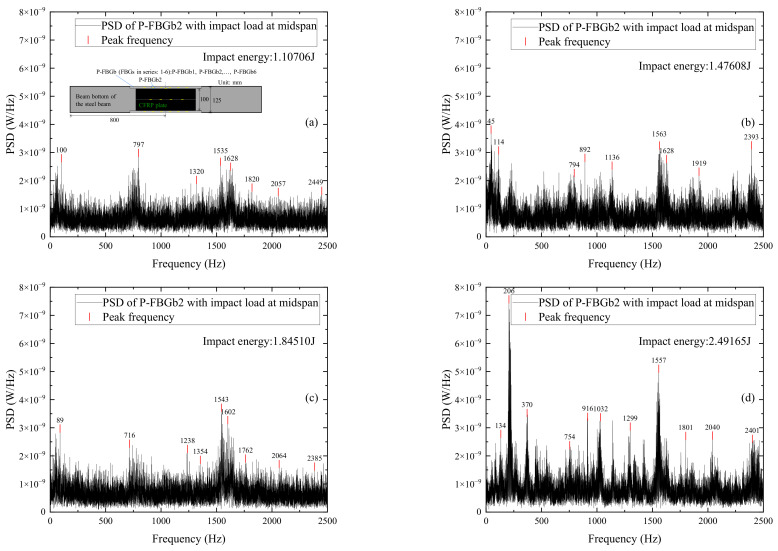
PSD distributions of signals measured by P-FBGb2 when the CFRP reinforced beam is subjected to different impact energies: (**a**) 1.10706 J; (**b**) 1.47608 J; (**c**) 1.84510 J; (**d**) 2.49165 J.

Figure 13 shows the power spectrum distributions of signals measured by P-FBGb2 when the CFRP reinforced beam is subjected to different impact energies. It can be seen from Figure 13 that although the peak values of the power spectrum at different impact energies vary, the peak frequencies at each impact condition are basically the same (i.e., the peak frequencies around 1500 Hz, 2000 Hz, and 2400 Hz), indicating that the CFRP reinforced beam has good impact resistance at each impact condition. Some extra peak frequencies in the individual diagrams may be due to differences in the impact action applied by the free-falling steel ball. A comparison of Figure 11 with Figure 13 shows that the peak signal of P-FBGd2 attached on the web is more pronounced than the peak signal of P-FBGb2 attached on the beam bottom when the beam is subjected to different impact energies.

**Figure 14:**
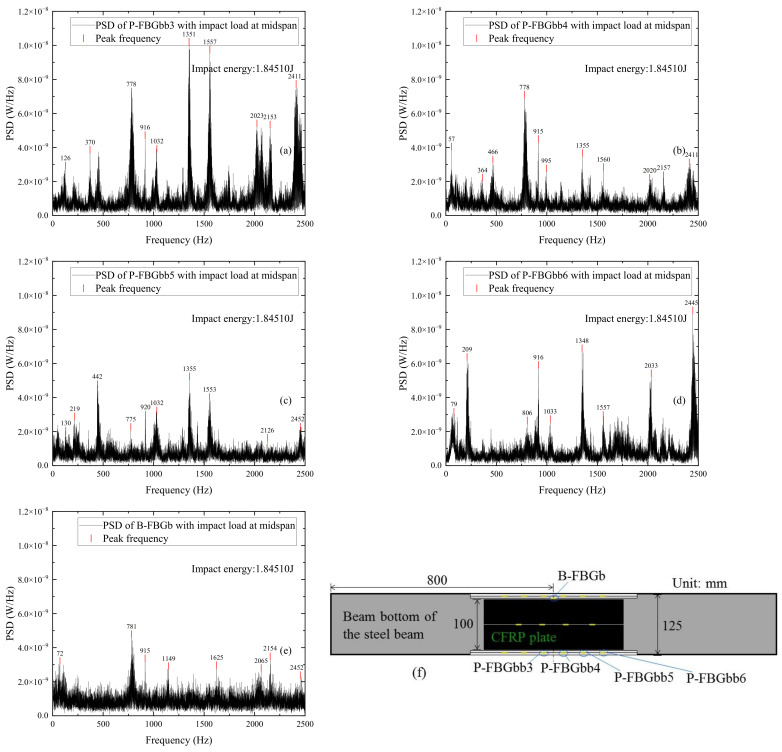
PSD distributions of signals measured by different FBG sensors when the CFRP reinforced beam is subjected to impact energy 1.8451 J: (**a**) P-FBGbb3; (**b**) P-FBGbb4; (**c**) P-FBGbb5; (**d**) P-FBGbb6; (**e**) B-FBGb; (**f**) Locations of the 5 FBGs.

Figure 14 shows the power spectrum distributions of signals measured by different FBG sensors when the CFRP reinforced beam is subjected to impact energy 1.8451 J. The FBG sensors include P-FBGbb3, P-FBGbb4, P-FBGbb5, P-FBGbb6, and B-FBGb on the beam bottom. It can be seen from Figure 14a–d that the peak signal of the FBG sensor closer to the impact point is more pronounced. Comparing Figure 14a–d with Figure 14e, it can be seen that the peak frequencies of the packaged FBG are basically the same as that of the bare FBG under equal impact energy. Since the location of P-FBGbb3 is closer to that of B-FBGb, a comparison on Figure 14a,e is performed. It shows that when the impact energy is the same, the peak signal of P-FBGbb3 is significantly higher than that of B-FBGb, indicating that the packaged FBG has better recognition of impact response than that of the bare FBG on the beam bottom. Comparing Figure 14e with Figure 12f, it can be seen that the peak signal of B-FBGb on the beam bottom is more pronounced than that of B-FBGd on the web.

### 4.3. FBG Sensors Embedded in the CFRP Plate

**Figure 15:**
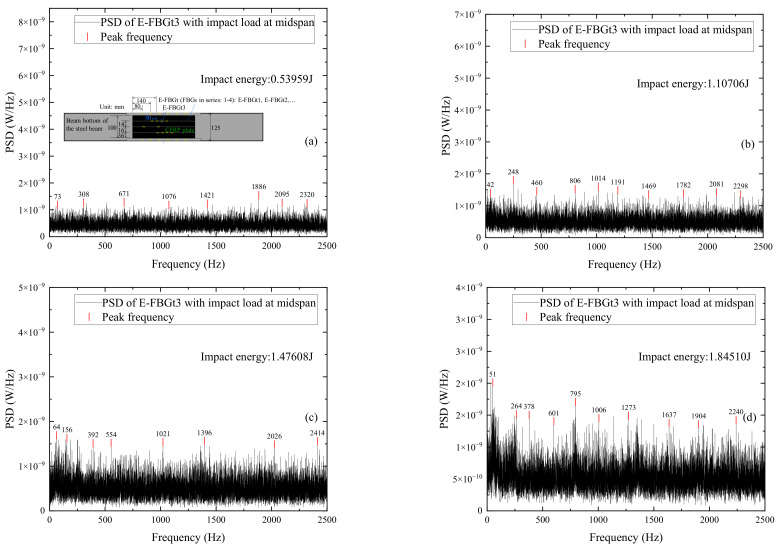
PSD distributions of signals measured by E-FBGt3 when the CFRP reinforced beam is subjected to different impact energies: (**a**) 0.53959 J; (**b**) 1.10706 J; (**c**)1.47608 J; (**d**) 1.8451 J.

It can be seen from the power spectrum that the peak value of the high-frequency component of the FBG sensors embedded in the CFRP plate is extremely insignificant and difficult to identify due to the influence of the DC component of the FBG signal in the frequency domain. Figure 15 shows the power spectrum distributions of signals measured by E-FBGt3 when the CFRP reinforced beam is subjected to different impact energies. It shows that the peak values of the high-frequency component under each impact action are not obvious, and the peak frequencies of the FBG sensors are partially identical.

**Figure 16:**
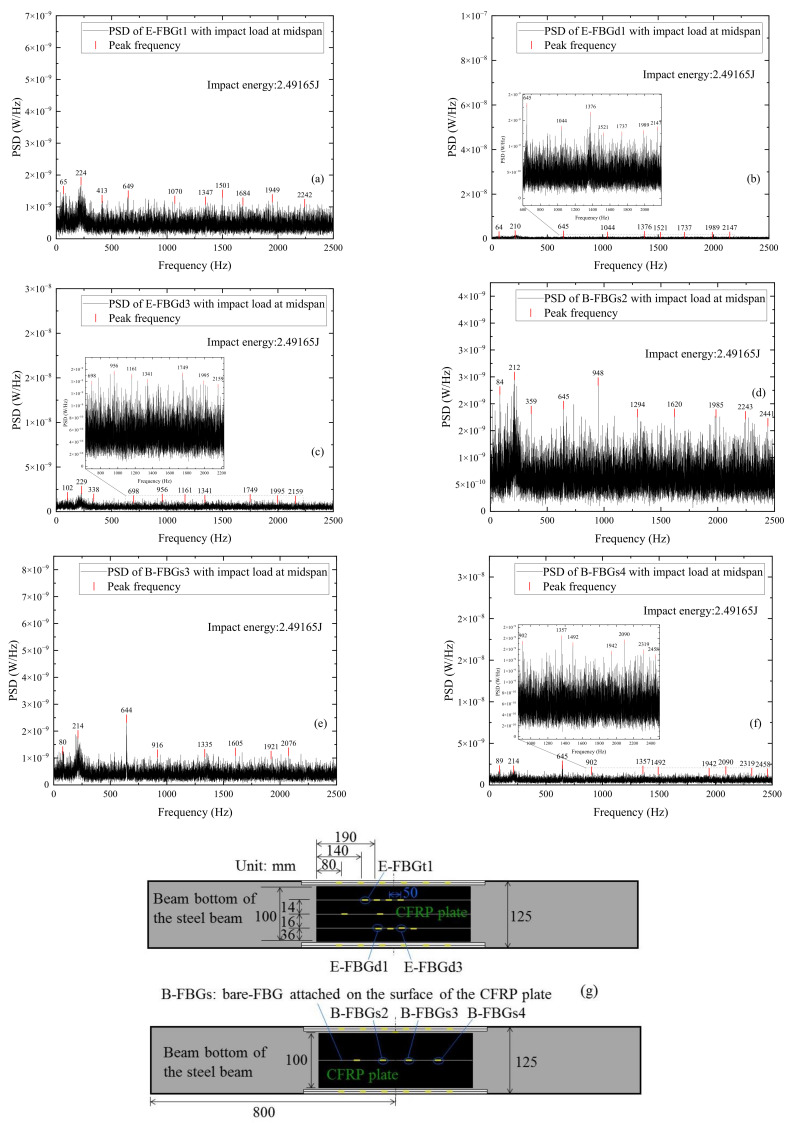
PSD distributions of signals measured by different FBG sensors when the CFRP reinforced beam is subjected to impact energy 2.49165 J: (**a**) E-FBGt1; (**b**) E-FBGd1; (**c**) E-FBGd3; (**d**) B-FBGs2; (**e**) B-FBGs3; (**f**) B-FBGs4; (**g**) Locations of the 6 FBGs.

It can be seen from the power spectrum that when the impact energy is small, the peaks of the high-frequency component of the FBG sensors embedded in the CFRP plate or externally attached to the surface of the CFRP plate are extremely inconspicuous. It can lead to difficult identification and then the case with the largest impact energy is selected for analysis. Figure 16 shows the power spectrum distributions of signals measured by different FBG sensors when the CFRP reinforced beam is subjected to impact energy 2.49165 J. The sensors include E-FBGt1, E-FBGd1, and E-FBGd3 embedded in the CFRP plate and B-FBGs2, B-FBGs3, and B-FBGs4 attached to the surface of the CFRP plate. Comparing Figure 16a–c with Figure 16d–f, it can be seen that when the impact energy is the same, the peak frequency of the bare FBG attached to the surface of the CFRP plate is slightly more obvious than that of the FBG embedded in the CFRP plate. The FBG embedded in the CFRP plate can still sense the external excitation and have good measurement accuracy, which can replace the surface-attached FBG sensors. Compared with the power spectrum of the FBG sensors attached to the web and beam bottom, the peak frequencies of the embedded and surface-attached FBG sensors in the CFRP plate are less pronounced. The reason may be that the steel beam shares most impact energy, while the attached CFRP plate is less efficiently utilized.

## 5. Conclusions

To develop effective health monitoring techniques for identifying the dynamic response of CFRP reinforced structures, impact testing of steel beam retrofitted with smart CFRP plates has been performed. Time and frequency domain analysis has been conducted to assess the dynamic performance of the reinforced structures and the sensitivity of the installed FBG sensors. Through the study, the following conclusions can be drawn:

(1) The amplitude of the wavelength increments of the FBG on the web increases with the growth of the impact energy, which validates the effectiveness of the proposed monitoring techniques. The CFRP reinforced steel beam shows good impact resistance during the impact testing, and no defect or damage has been identified from the time and domain analysis.

(2) The packaged FBG sensor is more effective than the bare FBG sensor in monitoring the external excitation-induced dynamic response of the CFRP reinforced steel beam, which is different from the static case. The FBG sensors embedded in the CFRP plate can replace the sensors attached to the surface of the CFRP plate to perceive the impact response.

(3) The peak values of the power spectrum at different impact energies vary. The peak frequencies transformed from the signals of FBG sensors attached on the web at each impact condition are basically identical (i.e., peak frequencies around 440 Hz, 1000 Hz, 1550 Hz, and 2200 Hz). The peak frequencies transformed from the signals of FBG sensors attached to the beam bottom at each impact condition are basically the same (i.e., the peak frequencies around 1500 Hz, 2000 Hz, and 2400 Hz). The slight difference can be attributed to the noise disturbance.

(4) The sensor used for temperature measurement cannot be embedded in the structure with a hollow pipe, which is different from the static case. For the temperature compensation of structures under dynamic load, it is suggested that the sensor merely for temperature measurement should be around the structure to avoid the possible vibration-induced dynamic deformation.

## Figures and Tables

**Table 1:** Cases for the impact action.

Item	Weight of Steel Ball (g)	Height of Impact Action (cm)	Impact Energy (J)
Case 1	110.12	20	0.21584
Case 2	261.02	20	0.51160
Case 3	376.55	20	0.73804
Case 4	508.5	20	0.99666
Case 5	110.12	30	0.32375
Case 6	261.02	30	0.76740
Case 7	376.55	30	1.10706
Case 8	508.5	30	1.49499
Case 9	110.12	40	0.43167
Case 10	261.02	40	1.02320
Case 11	376.55	40	1.47608
Case 12	508.5	40	1.99332
Case 13	110.12	50	0.53959
Case 14	261.02	50	1.27900
Case 15	376.55	50	1.84510
Case 16	508.5	50	2.49165

## Data Availability

The data supporting the results reported in the paper can be accessed from the corresponding authors.
